# Exploring the Potential of Genome-Wide Hybridization Capture Enrichment for Forensic DNA Profiling of Degraded Bones

**DOI:** 10.3390/genes16010023

**Published:** 2024-12-26

**Authors:** Christian Haarkötter, Xavier Roca-Rada, María Saiz, Diana C. Vinueza-Espinosa, Xiomara Gálvez, María Isabel Medina-Lozano, Daniel Díaz-Ruiz, Juan Carlos Álvarez, Bastien Llamas, Jose Antonio Lorente, Jeremy Austin

**Affiliations:** 1 Laboratory of Genetic Identification & Human Rights (LABIGEN-UGR), Department of Legal Medicine, Faculty of Medicine, University of Granada, PTS Granada, Av. Investigación 11, 18016 Granada, Spain; chaarkotter@ugr.es (C.H.); msaiz@ugr.es (M.S.); dvinuezaespinosa@ugr.es (D.C.V.-E.); xgales@ugr.es (X.G.); miml29@ugr.es (M.I.M.-L.); ddiazruiz1997@ugr.es (D.D.-R.); juanca@ugr.es (J.C.Á.);; 2 Australian Centre for Ancient DNA, The Environment Institute, School of Biological Sciences, The University of Adelaide, Adelaide, SA 5000, Australia; xavier.rocarada@adelaide.edu.au (X.R.-R.); bastien.llamas@adelaide.edu.au (B.L.); jeremy.austin@adelaide.edu.au (J.A.); 3 Faculty of Arts and Humanities, University of Coimbra, 3000-214 Coimbra, Portugal; 4 Australian Research Council Centre of Excellence for Australian Biodiversity and Heritage (CABAH), School of Biological Sciences, The University of Adelaide, Adelaide, SA 5000, Australia; 5 National Centre for Indigenous Genomics, John Curtin School of Medical Research, Australian National University, Canberra, ACT 0200, Australia; 6 Indigenous Genomics, Telethon Kids Institute, Adelaide, SA 5000, Australia

**Keywords:** ancient DNA, capture enrichment, human remains, massively parallel sequencing (MPS), Single Nucleotide Polymorphisms (SNPs)

## Abstract

In many human rights and criminal contexts, skeletal remains are often the only available samples, and they present a significant challenge for forensic DNA profiling due to DNA degradation. Ancient DNA methods, particularly capture hybridization enrichment, have been proposed for dealing with severely degraded bones, given their capacity to yield results in ancient remains. Background/Objectives: This paper aims to test the efficacy of genome-wide capture enrichment on degraded forensic human remains compared to autosomal STRs analysis. Methods: Six highly degraded human bones from the Spanish Civil War (1936–1939) were quantified with Quantifiler^™^ Trio and amplified with GlobalFiler^™^. Independently, partially UDG-treated double-stranded DNA libraries were generated and shotgun sequenced to screen for endogenous human DNA content. Subsequently, libraries were enriched with the Twist Bioscience “Twist Ancient DNA” reagent enrichment kit, which had not been previously tested for forensic purposes. Results: The results show that the samples behave similarly with both approaches (well-preserved samples yield good results). However, capture enrichment provides some new relevant insights, suggesting that its implementation in current NGS forensic platforms could be beneficial. Conclusions: Shotgun results show that the analyzed samples exhibit the same characteristics as ancient DNA samples in terms of DNA fragmentation and molecular damage, which may enhance the value of this approach when authenticating the endogenous DNA of forensic samples.

## 1. Introduction

Degraded human remains are probably one of the most challenging samples for a forensic genetics laboratory [1]. Following an individual’s death, the cessation of DNA repair mechanisms results in the continuous degradation of the genetic material [2], primarily due to hydrolytic and oxidative damage, the most significant contributors to DNA degradation [3]. These mechanisms are modulated by various environmental factors, including temperature, humidity, and acidic pH [4]. Still, in forensic contexts, such as disaster victim identification (DVI) or missing persons cases, teeth and bones often represent the sole biological material available [5].

In recent years, research has increasingly focused on massively parallel sequencing (MPS) technologies [6]. MPS is gaining prominence in forensic laboratories due to its versatile capabilities. This includes the analysis of degraded bones [7], as well as STR sequencing [8], mitochondrial genome analysis [9], Y-chromosome analysis [10], ancestry studies, and phenotypic inference [11]. This approach has been officially recognized by both the Scientific Working Group on DNA Analysis Methods (SWGDAM) [12] and the International Society for Forensic Genetics (ISFG) [13,14].

MPS technologies have also enhanced ancient DNA (aDNA) studies due to their capability of generating data for millions of SNPs from ultrashort DNA fragments [15]. Given the limited amount of endogenous DNA, target enrichment methods were developed so that sequencing could focus on particular regions of interest [16]. There are several commercial kits developed for target enrichment for ancient DNA, with the ones from Arbor Biosciences and Twist Bioscience being the most widely used [17], and based on the ‘1240k’ SNPs capture panel that targets 1.24 million autosomal SNPs [18]. The former, while presenting an allelic bias [19], has already been tested in forensic samples [20], but not the latter.

While having different goals—identification in the case of forensic DNA analysis and population genetics in the case of ancient DNA [21]—the incorporation of aDNA methods in forensic genetics has been discussed in the literature [22]. Hybridization enrichment has been tested in samples degraded by heat [23], for whole mitochondrial genome recovery with degraded skeletal remains [24], and for ancestry, phenotypic and Y chromosome SNPs with reference samples [25]. Additionally, it has been applied with an identity, sex chromosomes, ancestry, and phenotypic SNPs panel with mock degraded samples [26]. Nevertheless, to date, no tests have been conducted using the Twist Bioscience “Twist Ancient DNA” reagent enrichment kit with forensic samples.

The aim of this research is to explore the applicability of aDNA approaches in forensic samples in order to see if more information is obtained by testing the Twist Bioscience “Twist Ancient DNA” reagent enrichment kit, which has not been tested in the literature with this kind of application.

## 2. Materials and Methods

**Figure 1:**
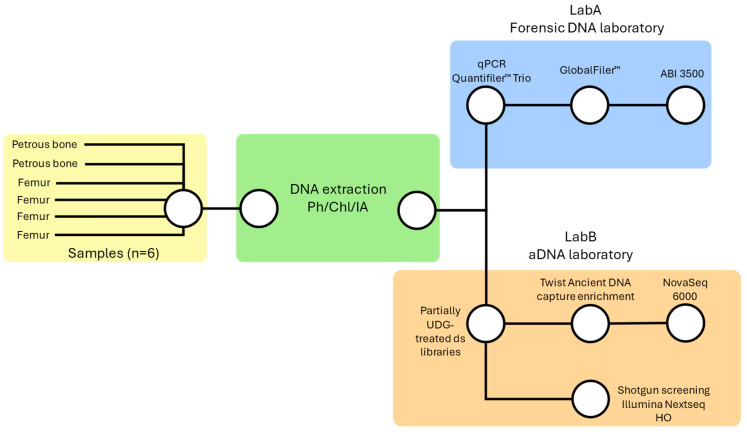
Description of the dual DNA analysis approach applied to the six bone samples. The methodology integrates both forensic (involving DNA extraction, Quantifiler^™^ Trio DNA quantification, GlobalFiler^™^ autosomal STRs amplification and capillary electrophoresis) and ancient (including partially UDG-treated double stranded libraries preparation, shotgun screening, Twist Ancient DNA capture enrichment, and deep sequencing) DNA methods. The above figure was created with Microsoft PowerPoint.

All of the pre-amplification procedures were conducted in dedicated facilities, adhering to the international recommendations for working with ancient DNA [27]. This entailed HEPA (High Efficiency Particulate Air) filtered positive pressure, UV room lighting sterilization, the use of sterilized material, and the use of clean room attires [28]. The general methodology is described in Figure 1.

### 2.1. Samples

Six human bone samples, as detailed in Table 1, were chosen for this study based on their autosomal STRs profile (see Methods Section 2.4 and Results Table 2): two with full profiles, two with partial profiles, and two with negative profiles. The samples were buried for 70–80 years in mass graves situated in Andalusia (Spain), in the context of the Spanish Civil War. The region is characterized by extreme temperatures, with maximum temperatures reaching 45 °C in summer and minimum temperatures of −3 °C in winter, along with slightly acidic soil pH [29,30]. The cochlear portion of the petrous bone or the medial diaphysis of the femur was sampled.

The bone samples were cut using a Dremel^®^ rotatory tool, and the resulting fragments were then irradiated in a UV cabin. The bone fragments were powdered using a TissueLyser^®^ II (QIAGEN, Hilden, Germany).

### 2.2. DNA Extraction

The DNA from the samples was extracted following an in-house protocol based on organic extraction [31]. In brief, one gram of bone powder was mixed with 5 mL of a lysis buffer (containing 0.5M EDTA, 10% SDS, 10 mg/mL proteinase K, and 1M DTT), and incubated overnight. The lysate supernatant was then mixed with 4 mL of phenol/chloroform/isoamyl alcohol (25:24:1), and the resulting supernatant was concentrated using Amicon^®^ Ultra-4 30kDa filters (Merck KGaA, Darmstadt, Germany). Finally, the extracts were purified using the MinElute^®^ PCR Purification kit (QIAGEN).

### 2.3. DNA Quantification

The DNA extracts were quantified using the QuantiFiler^™^ Trio Kit (ThermoFisher, Waltham, MA, USA) in accordance with the manufacturer’s recommendations [32] on a QuantStudio^™^ 5 (ThermoFisher).

### 2.4. DNA Amplification and Visualization

The quantified DNA extracts were amplified using the GlobalFiler^™^ commercial kit for autosomal STRs (ThermoFisher), following the recommended conditions [33]. A DNA input volume of 15 µL was used for each sample. Subsequently, capillary electrophoresis was conducted on an ABI 3500 Genetic Analyzer (ThermoFisher). The raw data were then analyzed using GeneMapper^™^ IDX v1.4. An analytical threshold of 50 RFU and a stochastic threshold of 360 RFU were established after internal validation [34].

### 2.5. Library Preparation and Enrichment

The same DNA extracts were partially UDG-treated [35], and double-stranded double-indexed libraries were generated [36]. In this step, a combination of uracil-DNA glycosylase and endonuclease VIII was used to remove all the uracils present in the ancient DNA, except for those located at the ends of the DNA fragments, which were to be detected as damage markers. Quality control and quantification of the generated libraries were performed using Qubit (ThermoFisher) and TapeStation (Agilent, Santa Clara, CA, USA). Libraries were sent for a screening shotgun sequencing using Illumina Nextseq HO (PE75) Sequencing Flowcell at the South Australian Genomics Centre (SAGC, Adelaide, Australia).

Libraries were subsequently over-amplified to reach 1000 ng in a PCR reaction with 5–10 µL of library, 25 µL of the KAPA HiFi HotStart ReadyMix (Roche, Basel, Switzerland), and 5 µL of 10 µM IS5 and IS6 primers, respectively. The PCR was set at 98 °C for 2 min, 15 cycles of 98 °C for 20 s, 56 °C for 30 s, 72 °C for 45 s, and 72 °C for 5 min. The reactions were purified using 1.2× AMPure XP beads (Beckman, Pasadena, CA, USA), and they were eluted in 30 µL of sterile water.

The enrichment reaction was performed using the Twist Ancient DNA commercial kit following the manufacturer’s protocol [37]. The panel used in the Twist ancient DNA capture enrichment reagent contains a core of 1240k SNPs used in population genetics analyses [18], including extra autosomal, X-chromosome, Y-chromosome, and phenotypic SNPs [37]. Post-enrichment PCR amplification was performed using the same polymerase and primers as described above, setting the PCR with one cycle of 95 °C for 24 s, 15 cycles of 98 °C for 15 s, 60 °C for 30 s, and 72 °C for 30 s, and a final extension of 72 °C for 60 s. Again, DNA purification was conducted with AMPure XP beads, and library quality control was conducted using Qubit and TapeStation.

Enriched libraries were sequenced using a NovaSeq 6000 System with a 2 × 100 bp SP Flow cell in the XP mode at the Kinghorn Centre for Clinical Genomics (Sydney, Australia).

### 2.6. Data Processing

Sequencing data were processed with the nf-core/eager version 2.4.6 aDNA analysis workflow package [38]. Merged reads were mapped to the GRCh37d5 reference genome using bwa aln with parameters-l 1024-n 0.01-o 2 [39]. The trimBam function of BamUtil was used for trimming the terminal ends of all retained reads, and standard quality filters (mapping quality ≥ q25 and base quality ≥ Q30) were applied using samtools v1.12 [40]. The reads were deduplicated using MarkDuplicates, and a pseudohaploid variant calling was performed with pileupCaller using the “Twist Ancient DNA” panel [17]. Ancient DNA authenticity (endogenous DNA percentage, fragment size, and post-mortem damage rate) was determined with DamageProfiler [41].

Mitochondrial DNA reads (length ≥ 30) were mapped to the revised Cambridge Reference Sequence (rCRS) [42] using the CircularMapper and bwa aln with the same parameters as that stated above (mapping quality ≥ q25 and base quality ≥ Q30). The read pileups were inspected in Geneious v2022.1.1, and mitochondrial haplogroups were called using HaploCart [43] and mitoverse HaploCheck 1.3.2 [44], that also calculated contamination estimates. Y chromosome haplogroups were called using HaploGrouper [45]. Kinship estimates were calculated using BREADR [46]. For the analysis of phenotypic variants (skin, eye, and hair color), only two samples met the quality thresholds necessary for random diploid variant calling using pileupCaller. These samples were genotyped using the SNP panel required by the HIrisPlex method to calculate the probabilities of phenotype prediction [47].

## 3. Results and Discussion

Both quantification data (human small target DNA quantity, human large target DNA quantity, human male target DNA quantity, degradation index, and DI) and the genetic profile data [number of alleles higher than the analytical threshold, the number of alleles higher than the stochastic threshold, relative fluorescence units (RFUs) and the number of reportable loci (homozygous markers that overcome the stochastic threshold and/or heterozygous markers that overcome the analytical threshold and show a peak height ratio ≥ 0.6] are shown in Table 2.

According to their performance, samples can be classified in three groups: a high DNA quantity and an almost complete genetic profile (samples 1 and 2), a low DNA quantity and a partial DNA profile (samples 3 and 4), and poor DNA quantity (fewer than 50 pg DNA) and a non-reportable profile (samples 5 and 6).

Shotgun and Twist capture results are shown in Table 3: number of mapped reads mapping filtering and PCR deduplication removal, endogenous DNA content, number of SNPs captured, SexDetermine results, and Mitochondrial DNA results (number of reads and haplogroup). All the sequencing data analysis results are presented in the Supplementary Materials section.

From all the SNPs contained in the Twist ancient DNA 1240k, there is variable overlap with the currently available forensic panels: 31% (39/124 SNPs) in the case of Precision ID Identity (ThermoFisher), 77% (127/164) in the case of Precision ID Ancestry (ThermoFisher), 60% (69/115) in the case of VISAGE Basic Tool, 49% (88/172) in the case of MAPlex, 51% (88/172) in the case of ForenSeq Signature (QIAGEN), 49% (80/162) in the case of ForenSeq Imagen (QIAGEN), and 67% (6874/10230) in the case of ForenSeq Kintelligence (QIAGEN) [48,49,50,51,52,53]. This means that, on average, 66% of the SNPs analyzed in the forensic field are present in the 1240k panel.

From the shotgun results, the same conclusions as the autosomal STRs are obtained regarding the behavior of the samples: one group with good sequencing results (samples 1 and 2), one group with average-low quality results (samples 3 and 4), and one group with poor results (samples 5 and 6). The mean length of the mapped reads after capture was 93.73 for sample 1, 95.34 for sample 2, 94.20 for sample 3, 97.99 for sample 4, 80.35 for sample 5, and 55.65 for sample 6, with the median for this last sample being 41.00. These results correspond to the results of both qPCR and autosomal STR profile: high sample DNA degradation (which translates into low quantification results and a ‘ski-slope’ genetic profile [54], particularly for samples 5 and 6).

**Figure 2:**
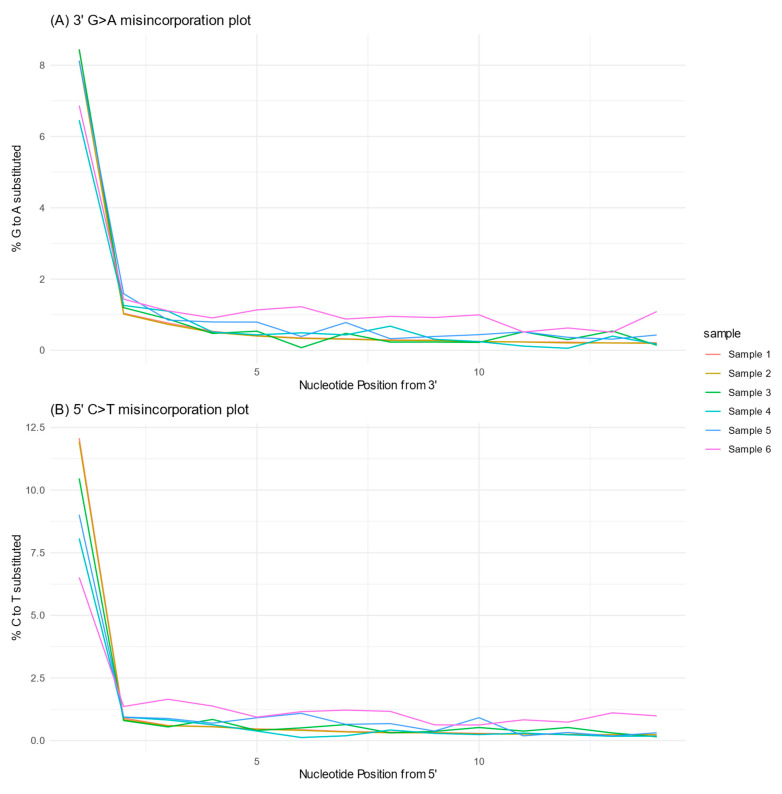
Analysis of DNA damage patterns at both 3′ (**A**) and 5′ (**B**) ends. The two plots show substitutions due to cytosine deamination, providing a view of the extent and nature of DNA damage in the samples studied. They illustrate the similarities between ancient DNA damage patterns and the analyzed forensic samples. The above figure was created in R using the MultiQC v1.13 output data.

In addition, new insights are obtained with the ancient DNA sequencing techniques, such as information about the molecular damage of the samples. Despite being considered as forensic samples, the analyzed samples show similar degradation and molecular damage patterns as ancient DNA samples: a low fragment size (50–60 bp on average) and cytosine deamination as typically observed when using the partial-repair library preparation method (see Figure 2). Moreover, no modern human DNA contamination was found (MOM and ML methods in shotgun results and mtDNA contamination estimates).

**Figure 3:**
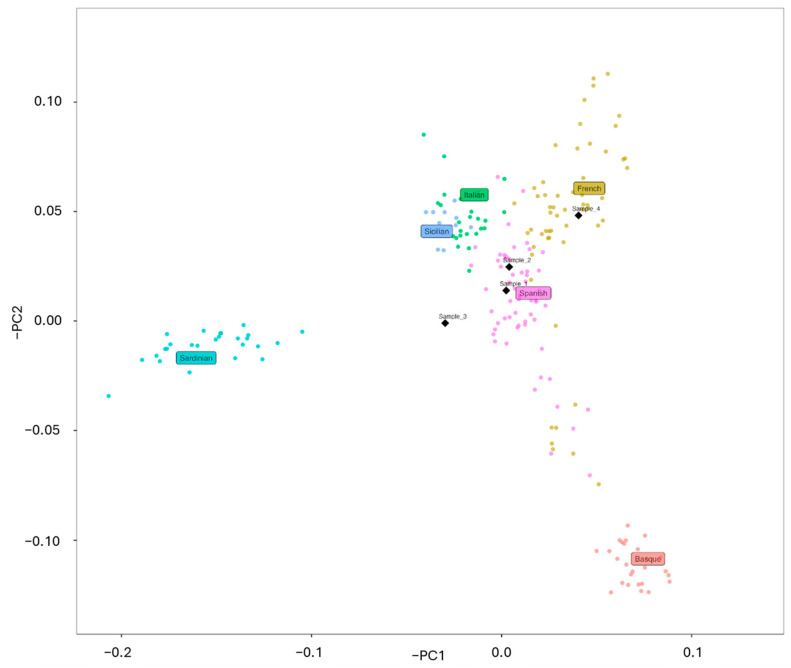
PCA of present-day Western Europeans, with projected forensic individuals as black diamonds. PCA was computed using smartpca software from the EIGENSOFT package (v7.2.1) with the lsqproject and SHRINKMODE option set to YES. Visualization was performed using R.

**Figure 4:**
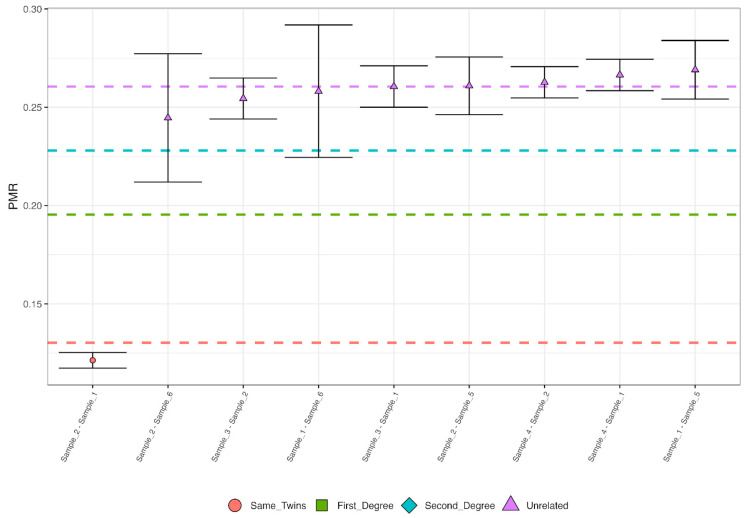
Kinship analysis using BREADR revealing unique relationship between samples 1 and 2. The pairwise mismatch rate (PMR) is shown on the *y*-axis, while pairs of individuals are displayed on the *x*-axis. Visualization was performed using R.

Interestingly, both mtDNA and Y chromosome haplogroups for these individuals are common in present-day Southern European populations, including Iberia, a finding supported by PCA (Figure 3) that suggested a possible Spanish ancestry for Sample 1, 2, and 3 as well as a possible French ancestry for Sample 4. The identification of identical haplotypes in Samples 1 and 2 suggested a possible kinship (Figure 4). The subsequent nuclear DNA analysis confirmed that the samples originated from the same individual, aligning with anthropological observations that documented the mass grave.

Of note, the number of mtDNA reads was unexpectedly low, as has been previously stated in an optimization study of the Twist capture kit [55], suggesting that both the quantity of mitochondrial probes and the number of PCR cycles should be optimized. The assigned mtDNA haplogroups were the same as those assigned by EMPOP (H1e1a6 MRCA 6.53 for Sample 1; H1e1a6 MRCA 2.67 for Sample 2; and H30b1 MRCA 6.57 for Sample 4), except for Sample 3, which was assigned to a different subclass (H2a2a MRCA 2.67).

The prediction of phenotypic is displayed in Table 4. Only the phenotypes of samples 1 and 2 could be predicted; however, the results are limited due to the missing SNPs. Brown eyes, light hair, and intermediate skin were the most likely phenotypic characteristics for these two samples.

**Figure 5:**
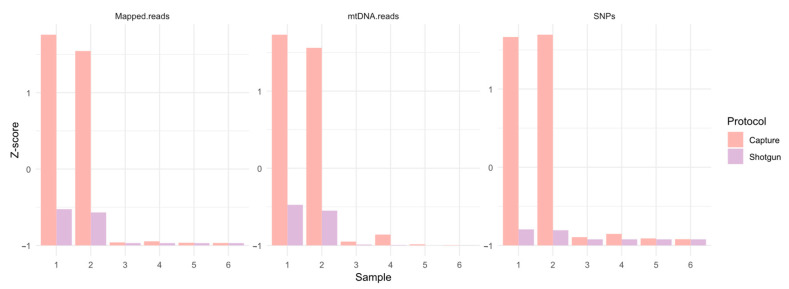
Z-scores comparison of the number of mapped reads, number of SNPs and mtDNA reads from the six samples with shotgun and with Twist capture. Visualization was performed using R.

The Twist capture protocol has never been tested in forensic samples before, yet it performed well here (see Figure 5). This improvement strongly supports the implementation of capture in the current NGS for forensic analyses [56]. The large number of SNPs recovered results in significant benefits for the forensic field after bioinformatic data analysis. Y chromosome haplogroups can be assigned with better resolution than when using STRs using tools like NevGen or Whit Athey. Additionally, mitochondrial DNA haplogroups, biological relationships between samples, and geographical origin can be determined. Finally, some phenotypic SNPs have been included in the TWIST capture assay, meaning that phenotypic characteristics can be inferred. All these features are obtained with a single reaction, whereas current forensic platforms require several separate kits for identification, genealogy, and phenotyping.

We also observed that libraries can be prepared and enriched with samples from which DNA has been extracted with phenol/chloroform/isoamyl alcohol, which is not the gold standard in ancient DNA protocols [31].

The ligation of oligonucleotides barcodes immediately following the DNA extraction and quantification presents a promising approach with potential applications in forensic genetics. This method could aid in controlling sample contamination, a concern of paramount importance in quality control and accreditation. This application has already been underscored in the literature [57].

Several studies have focused on degraded bone forensic DNA analysis, looking, in particular, for alternative methods to achieve better results with these challenging samples. For example, alternative sample sources, such as foot bones, have been proposed due to increased DNA yields [58]. Additionally, non-powder-based DNA extraction methods, such as large fragment demineralization, have been suggested [59]. The existing body of scientific literature on the application of ancient DNA techniques to forensic samples is relatively sparse, although the potential of ancient DNA techniques has already been emphasized in forensic literature [22]. As we will discuss below, different studies employ various panels, making comparisons among them challenging. This suggests that it would be beneficial for the scientific community to establish a set of forensically relevant SNPs, similar to the CODIS core loci [60].

Magnetic bead hybridization was used for the recovery of degraded forensic samples that were heated [23], and a protocol for in-solution hybridization for the whole mitochondrial genome enrichment was developed [61]. The MYTObaits kit for mitochondrial DNA enrichment was tested with degraded bones older than 70 years, improving the Sanger results and obtaining haplogroups from the majority of the samples [24]. Lastly, another study performed probe capture with the NimbleGen^®^ SeqCap EZ Probe Pool protocol in blood-derived DNA samples, with a custom panel of 135 identity SNPs, 40 ancestry SNPs, 24 phenotypic SNPs, 25 X chromosome SNPs, 81 Y-chromosome SNPs, 31 tri-allelic SNPs, 39 tetra-allelic SNPs, and 36 microhaplotype SNPs [26].

A recent study employed targeted enrichment (Arbor Bioscience myBaits kit) on burnt bones with different degrees of heat degradation. A custom panel was used, comprising a total of 3897 forensically relevant SNPS. These included identity SNPs, microhaplotype SNPs, phenotype-informative SNPs, ancestry SNPs, blood type SNPs, X chromosome SNPs, and Y chromosome SNPs. The study successfully classified Y haplogroups and estimated sexes for 56 out of the 86 sub-samples [20].

Another study performed with World War II (1939–1945) and Korean War (1950–1953) bone samples tested three library preparation methods, with and without DNA repair, and subsequent mitochondrial DNA capture, improving the genetic profile obtained with a purely forensic approach [62]. The study also concludes that their samples show the same degradation and molecular damage patterns as ancient DNA, so this kind of approach constitutes a valuable tool for dealing with these kinds of challenging samples.

A genetic genealogy panel (FORCE, FORensic Capture Enrichment) was developed with a core of 5400 identity, ancestry, phenotype, X and Y chromosomal SNPs, and a set of 3931 autosomal SNPs, using baits to target the selected SNPS, thereby recovering 44.4% SNPs in two 200-year-old bone samples [63]. Another custom panel of 164 SNPs (67 ancestry SNPs and 35 Y chromosome SNPs) was developed for hybridization enrichment for forensic samples, and successfully tested in buccal swabs [25].

Over the past few years, the potential for integrating ancient DNA techniques into the forensic field has been suggested, especially in relation to challenging samples such as degraded bones. Ancient DNA studies have achieved good results even with samples that are thousands of years old. This study aimed to test the applicability of capture enrichment in challenging forensic samples.

Firstly, we showed that the forensic samples exhibit the degradation and molecular damage characteristic of ancient DNA samples, i.e., cytosine deamination and short fragments (Figure 1; Table 3). This constitutes a valuable quality indicator and useful criteria for authenticating results. One could even implement a stringent bioinformatic pipeline by selecting only DNA molecules that exhibit signs of cytosine deamination [64] for downstream sequencing data analyses to ensure that potential contaminant DNA is not going to interfere with the results. This approach may be highly valuable in legal scenarios, as current forensic technologies cannot discriminate between authentic degraded DNA molecules and intact contaminant DNA molecules. Secondly, our study suggests that, despite not being a panacea for the analysis of challenging samples, capture enrichment constitutes a potential tool for implementation in forensic genetics. However, it has some disadvantages, such as the cost of the equipment and reagents, the hands-on time required for the procedures, and the complexity of data analysis (see Table 5). In this regard, we can only rely on advancements in the industry, which may provide an easy and automated process for library preparation, enrichment, and data analysis.

Thirdly, the potential of the generated data for analysis that can be performed should be noted, including kinship analysis (e.g., with the BREADR program for calculating the relatedness between samples with low coverage data [46]), population genetics (with programs such as smartPCA, based on principal components analysis) and phenotypic characteristics prediction. Genotype likelihoods can also be calculated with ANGsd package [65].

## 4. Conclusions

In the last decade, there has been an increasing interest in the potential applicability of ancient DNA techniques in forensic samples due to their ability to generate data from bones and teeth that are hundreds or even thousands of years old.

This study aimed to test the implementation of these techniques with actual challenging forensic samples. For this, the samples of six skeletal remains (two petrous bones and four femurs) were quantified with Quantifiler^™^ Trio, and autosomal STRs were amplified with Globalfiler^™^. Libraries were prepared from the same DNA extracts with the partial-repair method, and they were enriched and sequenced.

Shotgun results show that the analyzed samples exhibit the same characteristics as ancient DNA samples in terms of DNA fragmentation and molecular damage, which may enhance the value of this approach when authenticating the endogenous DNA of forensic samples.

## Figures and Tables

**Table 1:** Samples selected for the hybridization enrichment test.

Sample	1	2	3	4	5	6
Type	Petrous	Petrous	Femur	Femur	Femur	Femur
GlobalFiler^™^ Profile	Full	Full	Partial	Partial	Negative	Negative

**Table 2:** Quantification (Quantifiler^™^ Trio) and autosomal STRs amplification (GlobalFiler^™^) results.

Sample	QTRIO Small (ng/µL)	QTRIO Large (ng/µL)	QTRIO Y (ng/µL)	QTRIO DI	Alleles >AT	Alleles >ST	RFU	Loci
1	1.2260	0.0518	0.8391	24	31	27	5202	18
2	2.2091	0.1200	1.4168	18	37	33	7339	18
3	0.0040	0.0002	0.0039	20	20	7	382	10
4	0.0077	0.0008	0.0054	10	15	0	140	6
5	0.0026	0.0001	0.0022	26	8	0	113	3
6	0.0015	0.0002	0.0013	8	7	1	174	1

**Table 3:** Comparative analysis of shotgun (S) and capture (C) data. Y chromosome haplogroup and netScore and mitochondrial DNA haplogroups were determined using only captured data.

Sample	Mapped Reads	Endogenous DNA (%)	Mean Length Mapped Reads (bp)	SNPs (Out of 1,352,529)	Genetic Sex	Y Haplogroup	netScore	mtDNA Reads [1–16569]	mtDNA Haplogroup	Quality
1-S	2,115,402	25.20	66.06	54,610	M	E1b1b1b1a1c	919	634	H1e1a6	0.8848
1-C	12,956,742	41.52	93.73	1,107,558	M			3275		
2-S	1,915,737	23.57	66.13	50,059	M	E1b1b1b1a1c	923	543	H1e1a6	1
2-C	11,946,378	40.81	95.34	1,120,129	M			3071		
3-S	8537	0.14	61.78	194	M	R [K2b2a2]	9	17	H4a1	1
3-C	52,888	0.21	94.20	12,235	M			63		
4-S	6466	0.09	62.25	170	M	R1b1a1b	13	10	H30b1	0.7164
4-C	118,952	0.45	97,99	29,926	M			172		
5-S	7667	0.05	57.49	173	M	-	-	4	-	-
5-C	28,943	0.10	80.35	5232	M			21		
6-S	7435	0.02	47.37	133	M	-	-	5	-	-
6-C	16,164	0.03	55.65	876	M			7		

**Table 4:** DNA phenotyping report predicting eye, hair, and skin color using the HIrisPlex-S System.

		Sample 1	Sample 2
Eye color prediction	Predicted phenotype	Brown	Brown
	*p*-value	0.7983	0.9839
	AUC	0.9461	0.9461
	AUC loss	0.0048	0.0033
	Missing SNPs	1	1
Hair color prediction	Predicted phenotype	NA	NA
	*p*-value	NA	NA
	AUC	NA	NA
	AUC loss	NA	NA
	Missing SNPs	7	6
Hair shade prediction	Predicted phenotype	Light	Dark
	*p*-value	0.8001	0.7240
	AUC	0.9054	0.9054
	AUC loss	0.011	0.0092
	Missing SNPs	6	5
Skin color prediction	Predicted phenotype	Intermediate	Intermediate
	*p*-value	0.5001	0.5684
	AUC	0.7833	0.7833
	AUC loss	0.0233	0.0157
	Missing SNPs	14	13

**Table 5:** Five-star rating comparative analysis of three different DNA analysis technologies: capillary electrophoresis (CE), forensic next-generation sequencing platforms (NGS), and in-solution enrichment (aDNA). The accuracy of the results, time needed to obtain the results, sensitivity of the method, applicability to degraded samples, scalability, complexity of the workflow, and subsequent data analysis, integrability in a forensic laboratory, and cost are compared.

	CE	NGS	aDNA
Accuracy	★★★★★	★★★★☆	★★★★★
Time	★★★★★	★★★☆☆	★★☆☆☆
Sensitivity	★★★☆☆	★★★★☆	★★★★★
Degraded samples	★★☆☆☆	★★★☆☆	★★★★☆
Scalability	★★★★★	★★★★★	★★★★★
Workflow simplicity	★★★★★	★★★☆☆	★★☆☆☆
Data analysis ease	★★★★★	★★★☆☆	★☆☆☆☆
Integrability	★★★★★	★★☆☆☆	★☆☆☆☆
Cost	★☆☆☆☆	★★★☆☆	★★★★★

## Data Availability

Data is contained within the article or Supplementary Material.

## Supplementary Materials

The following supporting information can be downloaded at: https://www.mdpi.com/article/10.3390/genes16010023/s1. Supplementary Materials File: (Sheet 1) Shotgun screening MultiQC; (Sheet 2) Twist MultiQC; (Sheet 3) Twist mtDNA MultiQC; (Sheet 4) Haplogroups (mtDNA, Ychr); (Sheet 5) HIrisPlex.
